# Electrochemical aptasensor fabricated by anchoring recognition aptamers and immobilizing redox probes on bipolar silica nanochannel array for reagentless detection of carbohydrate antigen 15-3

**DOI:** 10.3389/fchem.2023.1324469

**Published:** 2023-12-13

**Authors:** Jun Xing, Qianqian Han, Jiyang Liu, Zhengzheng Yan

**Affiliations:** ^1^ Department of Breast Surgery, Shanxi Bethune Hospital, Shanxi Academy of Medical Sciences, Tongji Shanxi Hospital, Third Hospital of Shanxi Medical University, Taiyuan, China; ^2^ School of Chemistry and Chemical Engineering, Zhejiang Sci-Tech University, Hangzhou, China; ^3^ General Surgery Department, Shanxi Bethune Hospital, Shanxi Academy of Medical Sciences, Tongii Shanxi Hospital, Third Hospital of Shanxi Medical University, Taiyuan, China

**Keywords:** aptamer sensor, electrochemical analysis, reagentless detection, bipolar silica nanochannel array film, carbohydrate antigen

## Abstract

Timely, convenient, and efficient detection of carbohydrate antigen 15-3 (CA15-3) levels in serum holds significant importance in early screening, diagnostic assistance and prognosis prediction of breast cancer. The development of efficient and convenient electrochemical aptasensors with immobilized redox probes for label-free detection of CA15-3 is highly desirable. In this work, a bipolar silica nanochannel array film (bp-SNA) with two distinct functional domains including nanochannels and an outer surface was employed for the immobilization of recognition ligands and electrochemical redox probes, enabling the construction of a probe-integrated aptasensor for reagentless electrochemical detection of CA15-3. Cost-effective and readily available indium tin oxide (ITO) was used as the supporting electrode for sequential growth of a negatively charged inner layer (n-SNA) followed by a positively charged outer layer (p-SNA). The preparation process of bp-SNA is convenient. Functionalization of amino groups on the outer surface of bp-SNA was modified by aldehyde groups for covalent immobilization of recognition aptamers, further establishing the recognition interface. Within the nanochannels of bp-SNA, the electrochemical redox probe, tri (2,2′-dipyridyl) cobalt (II) (Co(bpy)_3_^2+^) was immobilized, which experienced a dual effect of electrostatic attraction from n-SNA and electrostatic repulsion from p-SNA, resulting in high stability of the immobilized probes. The constructed aptasensor allowed for reagentless electrochemical detection of CA15-3 ranged from 0.001 U/mL to 500 U/mL with a low detection limit (DL), 0.13 mU/mL). The application of the constructed aptasensor for CA15-3 detection in fetal bovine serum was also validated. This sensor offers advantages of a simple and readily obtainable supporting electrode, easy bp-SNA fabrication, high probe stability and good stability.

## 1 Introduction

Breast cancer is the most common malignant tumor in women, originating from breast tissue and typically occurring in the glands or ducts within the breast (Epaillard et al., 2023). Globally, millions of individuals are diagnosed with breast cancer each year. Early detection and comprehensive treatment are critical for improving survival rates and quality of life (Sullivan et al., 2021). Therefore, regular breast health examinations and medical follow-ups are essential for managing breast cancer. Tumor markers play a crucial role in early screening, diagnostic assistance and prognosis prediction of breast cancer (Rohanizadegan, 2018; Laraib et al., 2022). For instance, carbohydrate antigen 15-3 (CA15-3) is one of the most important biomarkers and can be used for early screening of breast cancer (Shahbazi et al., 2021). Commonly, elevated CA15-3 levels appear in the early stages of breast cancer. When used in conjunction with other diagnostic methods such as mammography, breast ultrasound, and magnetic resonance imaging, it helps doctors make more accurate diagnoses. After a breast cancer diagnosis, CA15-3 can be used to monitor disease progression and treatment efficacy. Regular monitoring of CA15-3 levels helps physicians assess treatment effectiveness and determine whether treatment plans need adjustment. Therefore, timely, convenient, and efficient detection of CA15-3 levels in serum holds significant importance.

So far, CA15-3 detection methods mainly include enzyme-linked immunosorbent assay (ELISA), radioimmunoassay (RIA), chemiluminescent immunoassay (CLIA), flow cytometry, and mass spectrometry (MS) (Jayanthi et al., 2017; Zhu et al., 2019; Pal et al., 2022). Specifically, ELISA and CLIA are commonly used for CA15-3 detection. They utilize antibody-based immunoassays and offer high sensitivity and specificity, making them suitable for routine screening and monitoring of breast cancer. RIA is not well-suited for large-scale screening owing to specialized equipment and safety precautions. Flow cytometry and MS are more suitable for research purposes as they require expensive equipment and specialized technical skills. Except for MS analysis, the above methods rely on antibody-based immunoassays (Filik and Avan, 2019). However, producing high-quality antibodies can be costly, and different batches of antibodies may exhibit variations, potentially leading to inconsistent results. Additionally, antibodies can lose their activity during storage or use, requiring specific storage conditions and maintenance. Developing new recognition elements and detection methods is crucial. Recently, aptasensors utilizing aptamers as specific recognition ligands have garnered significant attention (Anbiaee et al., 2023). Aptamers are artificially synthesized oligonucleotide molecules that can be precisely designed to bind to target molecules with high specificity. Aptamers offer excellent stability and are less prone to deactivation. They can be used under various environmental conditions without the need for specific storage conditions. Aptamers can be synthesized on a large scale at relatively low costs, making them suitable for large-scale applications. Furthermore, aptamers can be used to identify various molecular targets, including small molecules, proteins, nucleic acids, and more, rendering aptasensors applicable across a wide range of fields (Zhang et al., 2023; Domínguez-Renedo et al., 2023). Thus, developing novel aptasensors holds significant promise for efficient CA15-3 detection.

Electrochemical detection offers advantages such as simple instrument, fast operation, strong customizability, low cost, and real-time monitoring capability (Yan et al., 2022a; Yan et al., 2022b; Chang et al., 2022; Zheng et al., 2022). Electrochemical aptasensors provide powerful tools for applications in disease diagnosis, food safety monitoring, environmental monitoring, etc (Cui et al., 2014; Li et al., 2022; Desagani and Ben-Yoav, 2023). When using electrochemical aptasensors to detect CA15-3, a protein with no inherent electrochemical activity, it is necessary to monitor changes in the electrochemical signal of the redox probe when the aptamer binds to CA15-3 to achieve detection. Typically, the redox probe can either be free in the electrolyte solution or immobilized on the electrode surface. Amongst, electrochemical aptasensors based on the immobilization of redox probes on the electrode surface offer higher detection sensitivity (Maciel et al., 2022). This is attributed to the tight immobilization of the electrochemical redox probe on the electrode surface, resulting in a shorter electron transfer pathway. This detection mode also eliminates the potential impact of factors like dissolution, degradation, or oxidation on free probes. Compared to the extensive use of free probes, immobilized probes reduce reagent consumption, lower analysis costs, and enable real-time monitoring of target analytes without the need for constant probe addition. Therefore, the development of efficient and convenient electrochemical aptasensors with immobilized redox probes for label-free detection of CA15-3 is highly desirable.

Porous materials possess highly porous structures and a high specific surface area, finding extensive applications in the fields of biology and chemical analysis (Cui et al., 2020; 2021; Duan et al., 2021; Liu et al., 2022; Huang et al., 2023; Xu et al., 2023; Zhao et al., 2023). Utilizing porous materials to modify electrodes for probe immobilization can significantly enhance detection sensitivity and stability (Liu et al., 2020). On one hand, porous materials can dramatically increase the specific surface area of the electrode. This means that more immobilized probes can be loaded onto the electrode surface, improving probe immobilization efficiency and detection sensitivity (Chen et al., 2023). On the other hand, porous materials can be surface-functionalized to introduce specific chemical functional groups, enabling selective immobilization of different types of molecules, increasing the versatility of the sensor. Compared to porous nanoparticles, silica nanochannel film (SNA) has unique structure and characteristics. SNA possesses parallel, uniform, and size-tunable (typically 2–3 nm) nanochannel structures, displaying widespread applications in gas separation, liquid separation, catalytic reactions, sensing, drug delivery, nanofiltration, and more (Zhou et al., 2019; Su et al., 2022; Zhou et al., 2022; Zou et al., 2022; Zhang et al., 2023). For instance, due to the ultra-small nanochannels that impose size exclusion effects on proteins, DNA, and other large molecules or particles, SNA can be employed for the separation and analysis of biomolecules (Yang et al., 2022). Simultaneously, its surface carries a negative charge due to the low p*K*_a_ values of the silanol groups on SNA, causing ionization in commonly used electrolyte solutions. This results in charge-based permeability, allowing positively charged small molecules to pass through while repelling molecules with negative charges (Lv et al., 2022; Wei et al., 2022; Zhu et al., 2022). By modifying their surface groups to reverse the surface charge, this charge-based permeability can be altered accordingly (Gong et al., 2022; Cui et al., 2023). Furthermore, SNA can be conveniently prepared using a sol-gel reaction of siloxane precursors in the presence of surfactant micelles as templates (Walcarius et al., 2007; Teng et al., 2012). In addition, nanochannels and the outer surface of SNA can serve as independent modification regions (Chen et al., 2022; Huang et al., 2023). Combining its thin film structure with the matching dimension of two-dimensional (2D) planar electrodes, SNA-modified electrodes are expected to efficiently immobilize recognition aptamers and electrochemical probes, enabling the construction of probe-immobilized aptasensors for label-free, sensitive, and electrochemical detection of CA15-3.

In this work, a bipolar SNA (bp-SNA) composed of dual-layer SNA with opposite charges was modified on electrode surface to immobilize recognition aptamers and electrochemical probes, enabling the construction of an electrochemical aptasensor for reagentless detection of CA15-3. Tri (2,2′-dipyridyl) cobalt (II) (Co(bpy)_3_^2+^) with a low redox potential was employed as the electrochemical probe. To enhance the stability of the immobilized redox probe, negatively charged n-SNA and positively charged p-SNA were sequentially grown on the supported indium tin oxide (ITO) electrode. This allowed for stable immobilization of Co(bpy)_3_^2+^ based on electrostatic attraction from n-SNA and electrostatic repulsion from p-SNA. Following aldehyde derivatization of the amino groups on the outer surface of bp-SNA, covalent attachment of recognition aptamers was performed to establish an recognition interface. Upon recognition of CA15-3 by the aptamers, the increase in interfacial resistance resulted in a decrease in the electrochemical signal of the immobilized probe, enabling reagentless electrochemical detection of CA15-3. This electrochemical aptasensor based on immobilized probe offers advantages such as cost-effectiveness, probe stability, and low detection limit, making it highly promising for tumor biomarker detection.

## 2 Materials and methods

### 2.1 Chemicals and materials

Indium tin oxide (ITO) conductive glass electrodes (sheet resistance < 17 Ω/square, thickness: 100 ± 20 nm) were purchased from Kwei Tech Co., Ltd. (Zhuhai, China). Acidic calcium-binding protein (S100) was purchased from Okay Biotechnology (Nanjing, China). Carcinoembryonic antigen (CEA), carbohydrate antigen 15–3 (CA15-3), carbohydrate antigen 242 (CA242), cancer antigen 125 (CA125), prostate-specific antigen (PSA), and carbohydrate antigen 19–9 (CA19-9) were all procured from KeYue ZhongKai (Beijing, China). Amino-modified CA15-3 aptamer (5′-NH_2_- CTT​CCT​CCC​TGA​AGT​GAA​TAT​GAC​AGA​TCA​CAA​CTT​CCC​TCC​TTC -3′) was obtained from Sangon Biological Co., Ltd. (Shanghai, China). Tris(2,2′-bipyridyl) cobalt (II) hexafluorophosphate (Co (II) (bpy)_3_(PF_6_)_2_), 3-aminopropyltriethoxysilane (APTES), and potassium hydrogen phthalate (KHP) were purchased from Macklin Reagent Inc. (Shanghai, China). Bovine serum albumin (BSA) and hexaammineruthenium(III) chloride (Ru(NH_3_)_6_Cl_3_) were obtained from Sigma-Aldrich Trading Co., Ltd. (Germany). Potassium ferricyanide (K_3_[Fe(CN)_6_]), potassium ferrocyanide (K_4_[Fe(CN)_6_]), sodium chloride (NaCl), potassium chloride (KCl), glutaraldehyde (GA, 50% solution), cetyltrimethylammonium bromide (CTAB), sodium hydroxide (NaOH), sodium dihydrogen phosphate dihydrate (NaH_2_PO_4_·2H_2_O), glucose, tetraethyl orthosilicate (TEOS), disodium hydrogen phosphate dodecahydrate (Na_2_HPO_4_·12H_2_O), sodium nitrate (NaNO_3_) and ethanol were all purchased from Aladdin Bio-Chem Technology Co., Ltd. (Shanghai, China). Phosphate-buffered saline (PBS) was employed as the electrolyte solution for electrochemical tests, which was prepared by mixing NaH_2_PO_4_ and Na_2_HPO_4_ solution in a specific ratio. All chemicals and reagents used in this work were of analytical grade and used directly in the experiments. All solutions used during the experiments were prepared using ultrapure water (18.2 MΩ cm).

### 2.2 Characterization and instrumentations

The morphology of bp-SNA/ITO was examined using a transmission electron microscope (TEM, JEM-2100, JEOL, Japan). To prepare TEM samples, the bp-SNA layer was carefully scraped from the ITO electrode using a scalpel and dispersed in a minimal amount of ethanol, followed by ultrasonication for 0.5 h. Subsequently, the resulting dispersion was dropped onto a copper grid, dried under an infrared lamp, and then subjected to TEM observation with an acceleration voltage set at 200 kV. Cyclic voltammetry (CV), differential pulse voltammetry (DPV), and electrochemical impedance spectroscopy (EIS) tests were conducted using the Autolab electrochemical workstation (PGSTAT302N, Metrohm, Switzerland). All electrochemical tests used the standard three-electrode system, with either bare or modified ITO electrodes serving as the working electrode, platinum wire or foil as the counter electrode, and an Ag/AgCl electrode (saturated KCl solution) as the reference electrode.

### 2.3 Preparation of n-SNA modified ITO electrode

The modification of the ITO electrode with n-SNA was carried out using the Stöber solution growth method as reported in the literature (Teng et al., 2012). Specifically, CTAB (0.16 g) was dissolved in a mixture of ultrapure water (70 mL) and anhydrous ethanol (30 mL) with rapid stirring for 5 min. Then, ammonia (100 μL) and TEOS (80 μL) were added sequentially under stirring to prepare the precursor solution. Cleaned ITO electrodes (2.5 cm × 5 cm) were then immersed into the precursor solution to grow n-SNA at 60°C for 24 h. After the growth of n-SNA, the electrode was thoroughly rinsed with a large amount of ultrapure water and dried with nitrogen gas. Subsequently, the obtained electrode was aged at 100°C for 12 h. The resulting electrode contained surfactant micelles (SM) within the nanochannels, denoted as SM@n-SNA/ITO electrode. SM could be removed by immersing the electrode in a 0.1 M HCl/ethanol solution and stirring for 5 min. The electrode without micelles was referred to as the n-SNA/ITO electrode.

### 2.4 Fabrication of aptasensor with immobilized redox probe

The further growth of amino-functionalized p-SNA on the n-SNA/ITO surface was achieved using the electrochemical assisted self-assembly (EASA) method as reported in the literature (Walcarius et al., 2007; Li et al., 2023). Typically, ethanol (20 mL), NaNO_3_ (20 mL, 0.1 M, pH = 2.6) and CTAB (1.585 g) were mixed before APTES (318 µL) was added. After the pH was adjusted to ∼2.97 with concentrated HCl, TEOS (2372 µL) was added and stirred for 2.5 h at room temperature to obtained the precursor solution. Then, n-SNA/ITO electrode was immersed in the precursor solution and a constant current (−0.70 mA/cm^2^) was applied for 15 s. After the growth of p-SNA was completed, the electrode was rinsed thoroughly with a large amount of ultrapure water, dried with N_2_, and aged overnight at 100°C, resulting in the micelle-containing SM@bp-SNA/ITO electrode. To achieve the aldehyde derivatization of the amino groups on the outer surface of bp-SNA, the SM@bp-SNA/ITO electrode was immersed in a 5% GA solution in the dark for 30 min. Unbound GA was removed by rinsing with PBS (0.01 M, pH = 7.4) to obtain the GA/SM@bp-SNA/ITO electrode. After removing the micelles using an HCl-EtOH solution, the GA/bp-SNA/ITO electrode was obtained. The GA/bp-SNA/ITO electrode was immersed in a PBS solution (0.01 M, pH = 7.4) containing 1 mM Co(bpy)_3_^2+^ and stirred for 1 h to enrich the redox probe. After thoroughly rinsed with ultrapure water, GA/Co@bp-SNA/ITO electrode was obtained.

To construct the aptamer recognition interface, the GA/Co@bp-SNA/ITO electrode was immersed in a CA15-3 aptamer solution (0.4 μM in 0.01 M PBS, pH = 7.4) and incubated at 4°C for 1.5 h. Subsequently, it was thoroughly washed with PBS (0.01 M, pH = 7.4) to remove unbound aptamer and then dried. The electrode was then immersed in a BSA solution (0.1 wt%, dissolved in 0.01 M PBS, pH = 7.4) at room temperature and incubated for 30 min to block non-specific binding sites. After thorough rinsing, the aptamer sensor was obtained and denoted as Apt/GA/Co@bp-SNA/ITO.

### 2.5 Electrochemical detection of CA15-3

For CA15-3 detection, PBS (0.01 M, pH = 7.4) was used as the electrolyte solution. After incubating the aptamer sensor with different concentrations of CA15-3 at 4°C for 80 min (Wang et al., 2022), it was thoroughly rinsed with PBS (0.01 M, pH = 7.4). The changes in electrochemical signals before and after CA15-3 binding were measured. Using fetal bovine serum as a model, the analytical performance of the constructed aptamer sensor for real samples was investigated using the standard addition method. Before testing, the serum was diluted 50-fold with the electrolyte solution.

## 3 Results and discussion

### 3.1 The strategy for construction the probe-immobilized electrochemical aptasensor

**SCHEME 1:**
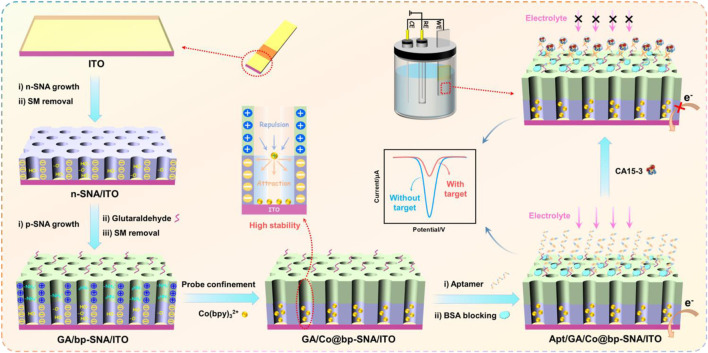
Illustration for the fabrication of probe-immobilized electrochemical aptasensor using bp-SNA modified ITO electrode.

In this work, bipolar SNA (bp-SNA) is used as an electrode modification layer for constructing a probe-immobilized electrochemical aptasensor. As shown in Scheme 1, the sensor fabrication process includes three aspects including the convenient preparation of bp-SNA, the fixation of electrochemical redox probes, and the immobilization of recognition ligands. Stöber solution growth and electrochemical-assisted self-assembly (EASA) are employed for preparing SNA. Both methods use sol-gel process of siloxane in presence of surfactant (e.g., cetyltrimethylammonium bromide, CTAB) micelles (SM). Commonly, the Stöber solution utilizes an alkaline ammonia/ethanol medium to control the sol-gel process of the siloxane precursor. On the other hand, EASA involves an acidic siloxane solution containing SM, where an appropriate negative voltage or current is applied to the electrode. Electrolysis of water on the electrode surface generates OH^−^, leading to a localized increase in pH, inducing the self-assembly of cationic surfactants and the condensation of siloxanes. The former allows for the one-time preparation of a large area of SNA-modified electrodes although time-consuming (e.g., 12 h). The latter offers rapid SNA preparation, such as completing SNA growth within 15 s. Herein, large-area ITO was used as the supporting electrode and the Stöber solution growth method was employed to prepare negatively charged n-SNA modified ITO electrodes in a one-step process, followed by electrode cutting. Therefore, a large quantity of n-SNA/ITO electrodes can be obtained simultaneously. Subsequently, amino-functionalized siloxanes were applied to further grow amino-modified and positively charged p-SNA on the n-SNA/ITO electrode using EASA method. It is worth noting that when SNA growth was complete, the nanochannels contain SM, which could be easily removed to obtain open nanochannel arrays.

Due to the dual functionality of bp-SNA, featuring separate domains for nanochannels and outer surface, it can be utilized as a substrate for constructing aptamer sensors with immobilized probes. Specifically, the outer surface is employed for anchoring recognition aptamers, while the nanochannels serve as a platform for immobilizing redox probes. As shown in Scheme 1, the amino groups on the outer surface of bp-SNA can be derivatized with aldehyde groups for covalent immobilization of recognition aptamers. To achieve directed derivatization on the outer surface of bp-SNA while avoiding cross-linking of amino groups within the nanochannels, aldehyde derivatization was carried out before the removal of SM. Due to the presence of SM, which blocked the nanochannels, the reaction between the bifunctional reagent (GA) and amino groups occurs exclusively on the outer surface (GA/SM@bp-SNA/ITO). Subsequently, after removing SM to obtain an array of open nanochannels (GA/bp-SNA/ITO), the redox probe Co(bpy)_3_^2+^ was immobilized within the nanochannels through stirring (GA/Co@bp-SNA/ITO). Following this, recognition aptamers (Apt) were covalently anchored on the outer surface of bp-SNA. After blocking non-specific sites with BSA, the aptasensor was obtained (Apt/GA/Co@bp-SNA/ITO).

Since Co(bpy)_3_^2+^ was immobilized on the electrode surface, the aptamer sensor will exhibit its redox signal. When the solution contained the analyte, CA15-3, the complex formed by the aptamer and CA15-3 will increase the electrode interface resistance and hinder the diffusion of the supporting electrolyte to the underlying electrode, thus reducing the electrochemical signal of Co(bpy)_3_^2+^. Based on this signal turn-off mechanism, electrochemical detection of CA15-3 can be achieved.

### 3.2 Characterization of the bp-SNA/ITO electrode

**FIGURE 1:**
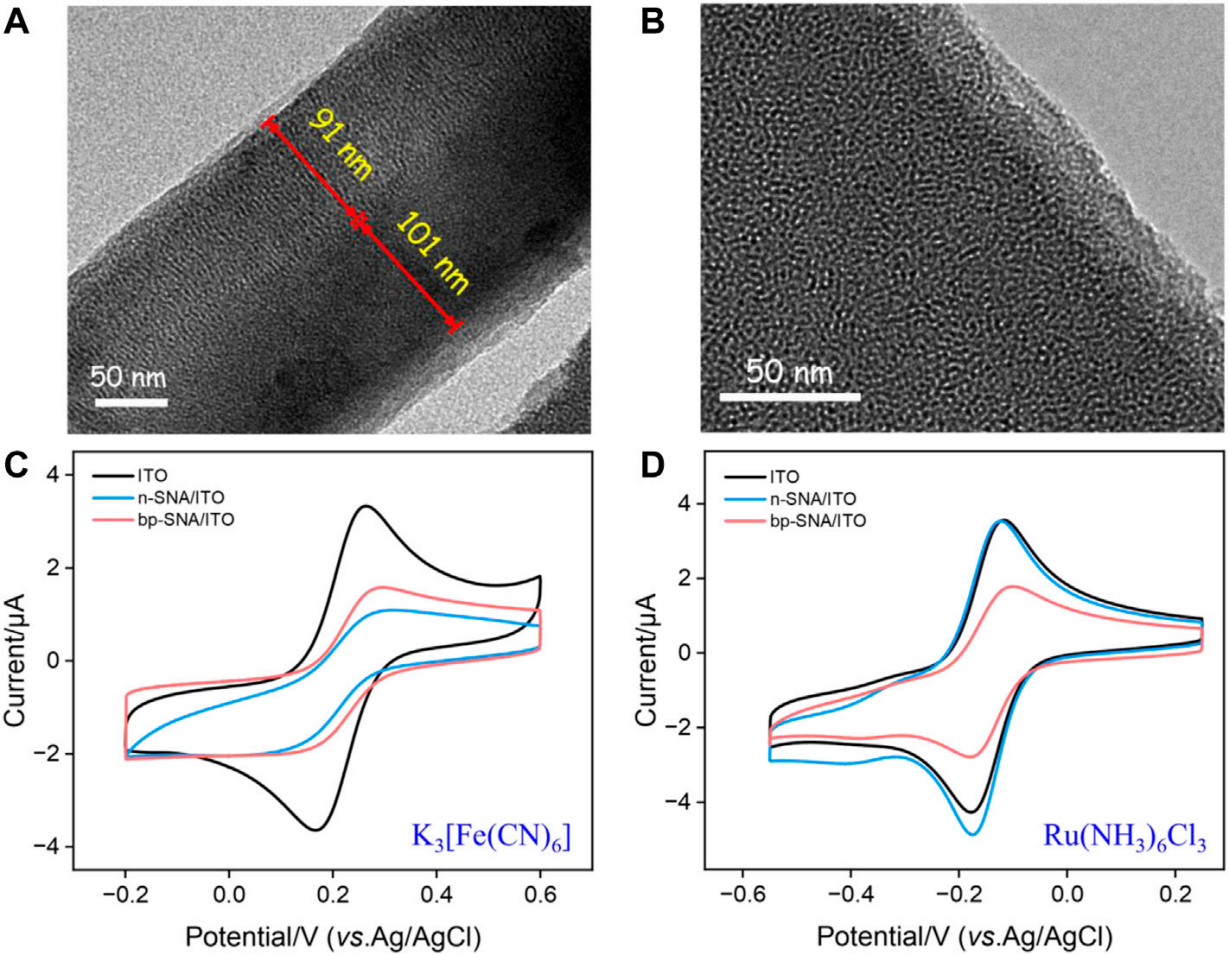
**(A)** TEM image of the cross-sectional bp-SNA. **(B)** Top-view TEM image of bp-SNA. **(C,D)** CV curves on different electrodes in Fe(CN)_6_^3−^ (50 μM, **(C)** or Ru(NH_3_)_6_^3+^ (50 μM, **(D)** in KHP (50 mM, pH = 4).

The morphology and thickness of bp-SNA were characterized using transmission electron microscopy (TEM). As shown in Figure 1A, the cross-sectional TEM image clearly revealed the double-layer SNA structure of bp-SNA. Each layer was composed of parallel-aligned nanochannels. The outer p-SNA layer had a thickness of 91 nm, while the inner n-SNA layer was 101 nm in thickness. Figure 1B presented a top-view TEM image, where each bright spot represented a nanochannel structure. As observed, the entire film exhibited no noticeable defects, and the nanochannels were uniformly arranged with diameters distributed between 2 and 3 nm.

To confirm the successful preparation of the bp-SNA/ITO electrode and the related charge-selective permeability, the electrochemical signals of the standard electrochemical redox probes on different electrodes were measured using cyclic voltammetry (CV). Figures 1C, D compared the CV curves of ITO, n-SNA/ITO, and bp-SNA/ITO electrodes in solutions of the anionic probe Fe(CN)_6_^3−^ or the cationic probe Ru(NH_3_)_6_^3+^. From Figure 1C, it can be observed that the peak current of Fe(CN)_6_^3−^ significantly decreased compared to bare ITO after modification with n-SNA. This is attributed to the silica structure of n-SNA, which makes its surface rich in silanol groups with low p*K*a (∼2). Consequently, the ionization of silanol groups in the test solution generated a negatively charged surface, resulting in electrostatic repulsion towards Fe(CN)_6_^3−^. When further modified with p-SNA on the electrode, the protonation of amino groups created positive charge sites that can electrostatically adsorb Fe(CN)_6_^3−^. Thus, the signal peak of Fe(CN)_6_^3−^ on the bp-SNA/ITO electrode was slightly increased compared to the n-SNA/ITO electrode. When the electrochemical probe used was the cationic Ru(NH_3_)_6_^3+^, the electrostatic attraction between n-SNA and Ru(NH_3_)_6_^3+^ slightly increases the electrochemical signal on n-SNA/ITO electrode (oxidation/reduction peak current: 3.485 μA/−4.572 μA) compared to bare ITO (oxidation/reduction peak current: 3.357 μA/−4.013 μA). However, on the bp-SNA/ITO electrode, Ru(NH_3_)_6_^3+^ experiences a synergistic effect of electrostatic repulsion from the outer p-SNA layer and electrostatic attraction from the inner n-SNA layer, leading to a decrease in peak current. These results demonstrate the charge-selective permeability of bp-SNA.

### 3.3 Stable confinement of Co(bpy)_3_^2+^ on the bp-SNA/ITO electrode

**FIGURE 2:**
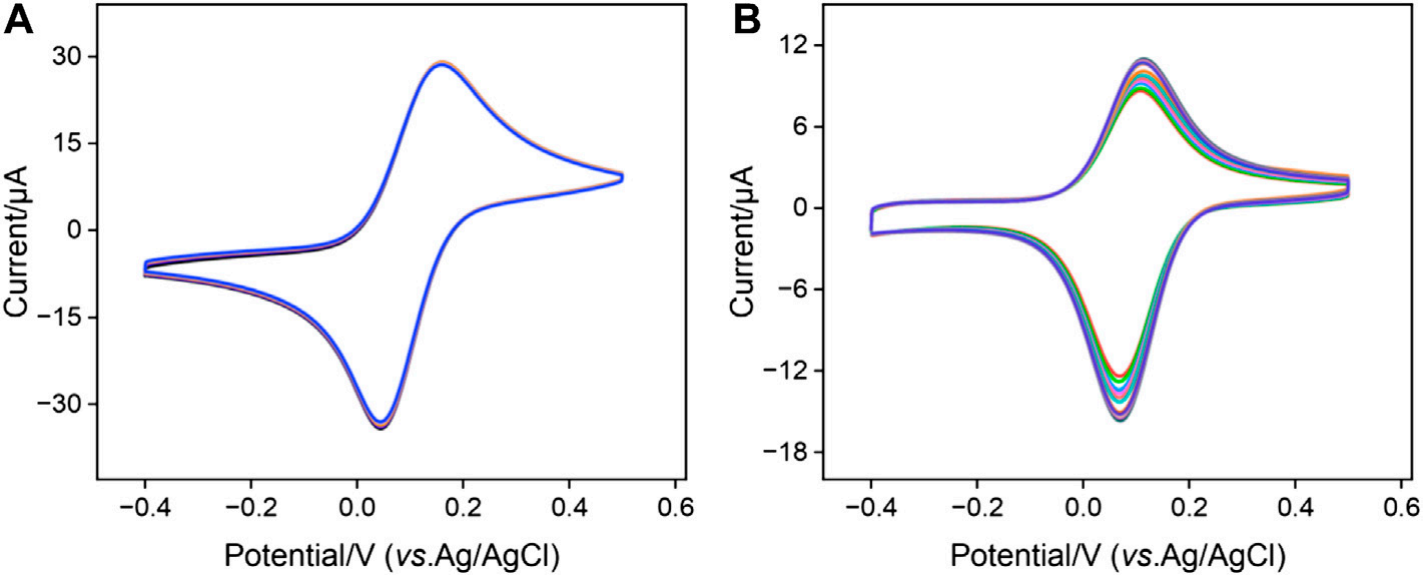
Successive 20-turn CV curves of **(A)** Co@bp-SNA/ITO or **(B)** Co@n-SNA/ITO electrodes in PBS (0.01 M, pH = 7.4) at a scan rate of 50 mV/s.

The stability of positively charged probe Co(bpy)_3_^2+^ within the nanochannels of the bp-SNA/ITO electrode is a critical for the performance of aptamer-based sensors. The electrochemical stability of the Co@bp-SNA/ITO electrode was evaluated by conducting 20 consecutive CV scans in a PBS (0.01 M, pH = 7.4) solution. Signal of Co@n-SNA/ITO was also measured under the same conditions as a control. As shown in Figure 2A, after 20 consecutive scans, the signal of the Co@bp-SNA/ITO electrode remained unchanged, indicating that there was no significant leakage of the Co(bpy)_3_^2+^ probe during the testing process. This excellent stability can be attributed to the dual electrostatic forces exerted by bp-SNA on Co(bpy)_3_^2+^, comprising the electrostatic attraction of the inner n-SNA to the probe and the electrostatic repulsion from p-SNA.

In contrast, as depicted in Figure 2B, the peak current of Co@n-SNA/ITO gradually decreased with an increase in the number of scan cycles under the same conditions, with a 25% reduction in the electrochemical signal after 20 cycles compared to the initial value. This suggests that Co(bpy)_3_^2+^ diffused into the solution during continuous scanning due to concentration polarization, indicating that n-SNA cannot achieve stable immobilization of Co(bpy)_3_^2+^. Therefore, the utilization of bp-SNA for stable confinement and immobilization of Co(bpy)_3_^2+^ offers a convenient film growth process and high signal stability, providing a novel approach for constructing solid-state electrochemical sensing platforms.

### 3.4 Characterization of the fabrication process of electrochemical aptasensor

**FIGURE 3:**
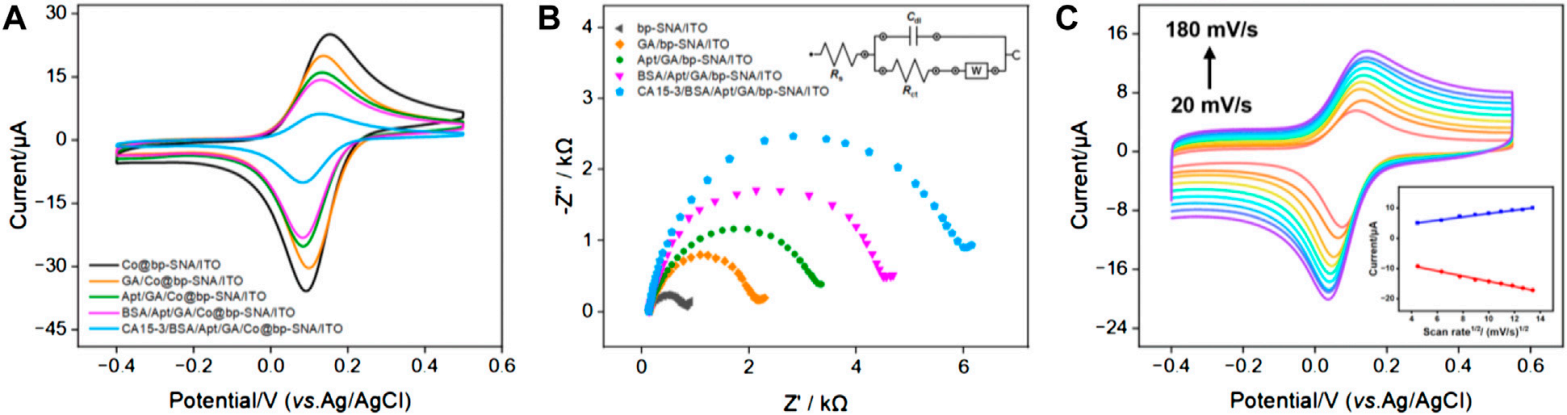
**(A)** CV curves obtained on different electrodes in PBS (0.01 M, pH = 7.4). **(B)** EIS plots obtained from different electrodes in 0.1 M KCl containing 2.5 mM Fe(CN)_6_^3−/4−^. The inset diagram represents the equivalent circuit. **(C)** CV curves obtained on the aptasensor in PBS (0.01 M, pH = 7.4) at different scan rates. Insert is the linear relationship of anodic and cathodic peak currents on the square root of scan rate.

Changes at the electrode interface during the construction of the aptasensor were studied using CV and electrochemical impedance spectroscopy (EIS). Figure 3 displayed the CV and EIS curves for different electrodes in PBS solution. As shown in Figure 3A, compared to the electrode directly immobilizing the redox probe without prior GA derivatization (Co@bp-SNA/ITO), the electrode where GA reacted with the amino groups on the outer surface of bp-SNA before immobilizing the electrochemical probe (GA/Co@bp-SNA/ITO) exhibited a slight reduction in the redox peak. This reduction may be attributed to changes in the electrode interface resistance resulting from the reaction between GA and the amino groups. After covalently attaching the aptamer to the GA/Co@bp-SNA/ITO electrode surface, the redox peak current further decreased, indicating a significant increase in interface resistance due to the insulating nature of the protein. Furthermore, after BSA blocked non-specific sites on the electrode surface, the redox peak of the immobilized probe continued to decrease. Additionally, after incubation of the receptor sensor with CA15-3, the electrochemical signal significantly decreased, confirming the successful formation of the aptamer-antigen complex at the sensing interface.

The EIS plot obtained on different electrodes were shown in Figure 3B. The equivalent diameter of the semicircular portion in the high-frequency region corresponded to the apparent charge transfer resistance (*R*_ct_). As the electrode surface was gradually modified, the semicircular diameter in the high-frequency region of EIS gradually increased, suggesting an increasing *R*_ct_, which demonstrated an increase in electronic transfer resistance at the electrode interface. These results further confirmed the successful construction of the bio-interface during sensor fabrication and recognition processes. Figure 3C displayed the CV curves obtained on CA15-3/BSA/Apt/GA/Co@bp-SNA/ITO electrode at different scan rates in 0.01 M PBS (pH 7.4). As the scanning speed increased, the peak current also increased. As revealed in inset of Figure 3C, both the anodic peak currents (*I*_pa_) and cathodic peak currents (*I*_pc_) exhibit a linear relationship with square root of the scan rate (*v* ^1/2^; *I*_pa_ = 2.68 *v* ^1/2^ + 0.548, *R* ^2^ = 0.992; *I*_pc_ = −5.63 *v* ^1/2^–0.859, *R* ^2^ = 0.994), suggesting a diffusion-controlled electrochemical process. This phenomenon proved that the Co(bpy)_3_^2+^ probe electrostatically confined into the nanochannels was still diffusive in the nanospace.

### 3.5 Optimization the conditions for the fabrication of aptasensor

**FIGURE 4:**
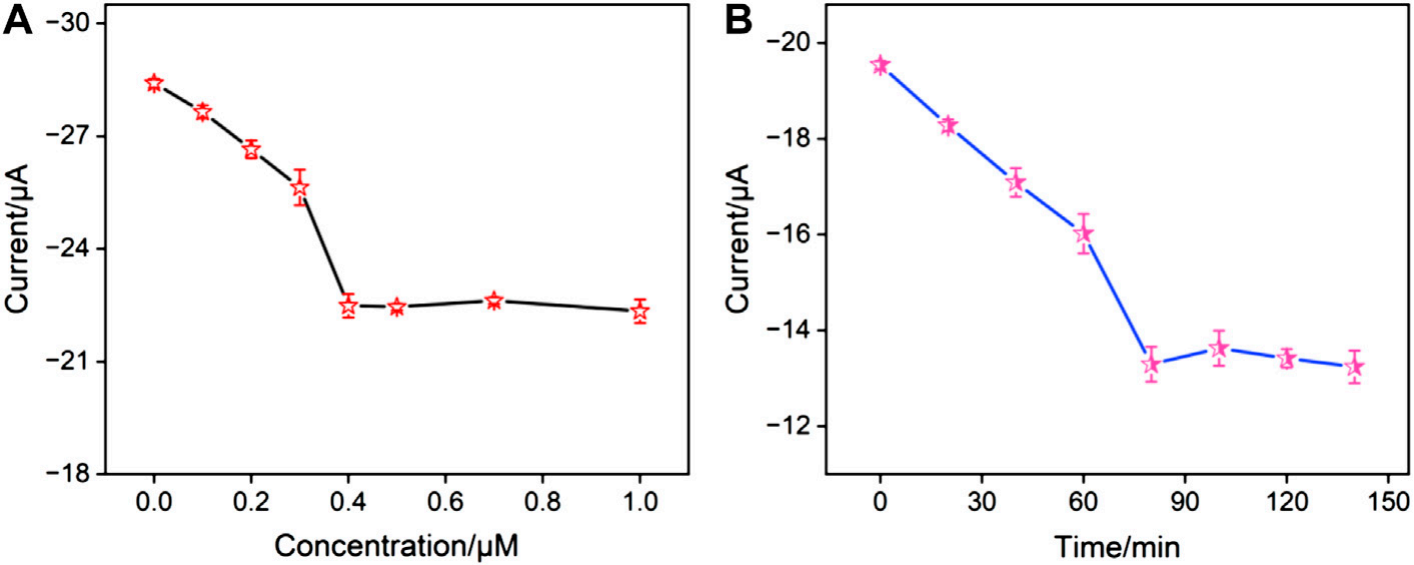
**(A)** The peak currents obtained on aptasensor fabricated using different concentration of aptamer. **(B)** The peak currents obtained after the aptasensor incubated with CA15-3 for different time.

To achieve the best detection performance, conditions for the fabrication of the aptasensor were optimized, including the concentration of the aptamer used to fabricate the sensing interface and the incubation time with CA15-3. Figure 4A presented the peak current of the electrodes obtained using different concentrations of the aptamer for preparing the sensing interface. It can be observed that when the aptamer concentration was 0.4 μM, the electrochemical signal of the electrode stabilized, indicating that at this concentration, the fixed amount of the receptor on the sensing interface had reached saturation. Figure 4B showed the electrochemical signal of the aptasensor after incubation with CA15-3 for different incubation time. It can be seen that after 80 min of incubation, the electrode signal became stable, confirming that the binding had reached saturation. Thus, these parameters were chosen for subsequent experiments.

### 3.6 Electrochemical reagentless detection of CA15-3

**FIGURE 5:**
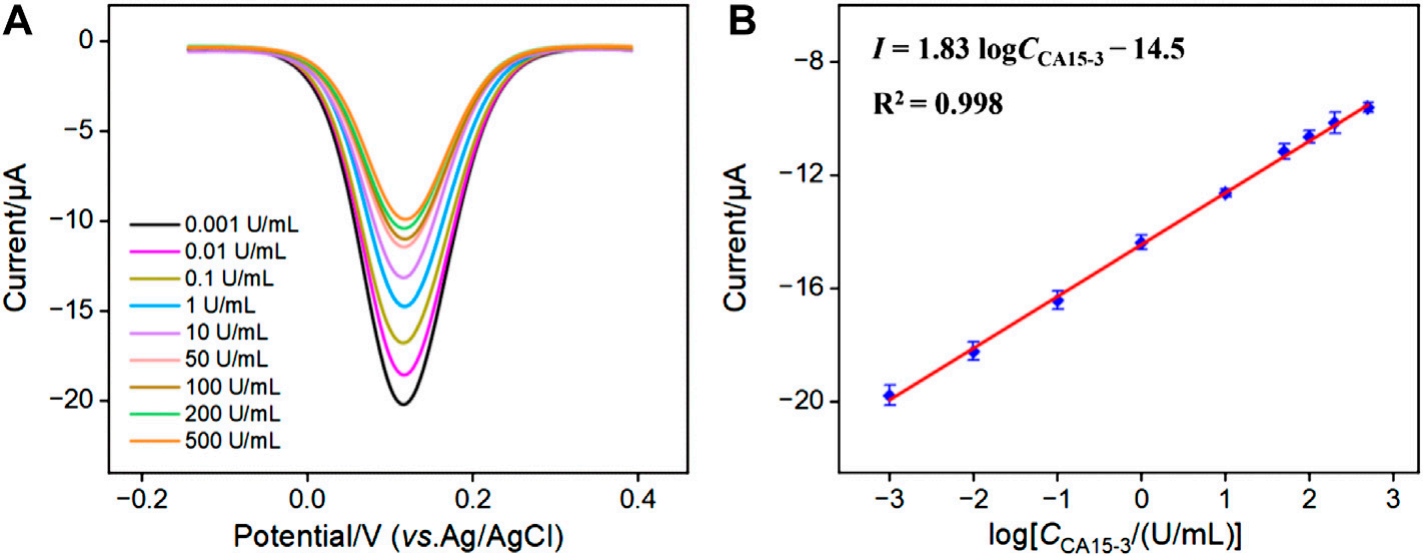
**(A)** DPV curves for detecting different concentrations of CA15-3 using the developed aptasensor. **(B)** The corresponding linear calibration curve.

Figure 5A depicted the differential pulse voltammetry (DPV) curves of the aptasensor after incubation with different concentrations of CA15-3. It is evident that as the CA15-3 concentration increased, the reduction peak current of the immobilized probe decreased. This was attributed to the formation of a complex between the receptor and CA15-3, leading to an increase in interface resistance. Figure 5B shows the corresponding linear regression line. It can be observed that the reduction peak current (*I*) of the probe exhibited a good linear relationship with the logarithm of CA15-3 concentration (log*C*_CA15-3_) (*I* = 1.83 log*C*_CA15-3_ − 14.5, *R* ^2^ = 0.998). The detection limit (DL) calculated based on a signal-to-noise ratio (S/N) of 3 was 0.13 mU/mL. Table 1 summarized the comparison of the analytical performance obtained on different modified electrodes for the detection of CA15-3 using electroluminescence (ECL), photoelectrochemical (PEC) or electrochemical (EC) methods (Cui et al., 2014; Bao et al., 2021; Zhong et al., 2021; Kongkaew et al., 2023; Lin et al., 2023). As seen, the electrochemical aptasensor established in this paper has a wide linear range and a low LOD. In addition, the fabrication of the aptasensor avoided complicated steps for synthesis of complex material or electrode modification.

**TABLE 1:** Comparison of CA15-3 detection performance using different methods.

Electrode	Sensor	Construction step	Probe mode	Linear range (U/mL)	LOD (mU/mL)	Ref.
Ab/GA/PPY-luminol-AuNPs/ITO	ECL immunosensor	6	Free	0.001–700	0.58	Bao et al. (2021)
Ab/CeO_2_/Pt/rGO/NH_2_-SiO_2_-PTCA/GCE	ECL immunosensor	7	Immobilized	0.012–120	1.4	Lin et al. (2023)
Apt/Zn-MOF/GCE	PEC aptasensor	6	Immobilized	0.05–100	28	Zhong et al. (2021)
Ab/Cryogel-PBNCs/MEA	EC immunosensor	9	Immobilized	0.0005–0.012	0.49	Kongkaew et al. (2023)
M-Pt-Ab_2_/Ag/Ab_1_/GS/SPCE	EC immunosensor	6	Free	0.008–21	1.0	Cui et al. (2014b)
Apt/GA/Co@bp-SNA/ITO	EC aptasensor	4	Immobilized	0.001–500	0.13	This work

PPY, polypyrrole; AuNPs, gold nanoparticles; ITO, indium-tin oxide; ECL, electrochemiluminescence; PTCA, 3,4,9,10-perylenetetracarboxylic acid; GCE, glassy carbon electrode; Zn-MOF, metal-organic framework; PEC, photoelectrochemical; PBNCs, prussian blue nanocubes; MEA, multi-electrode array; M-Pt, mesoporous platinum nanoparticles; Ab_2_, the secondary antibody; Ab_1_, the first antibody; GS, graphene sheet; SPCE, screen printed carbon electrode.

### 3.7 Selectivity, stability and repeatability of the aptasensor

**FIGURE 6:**
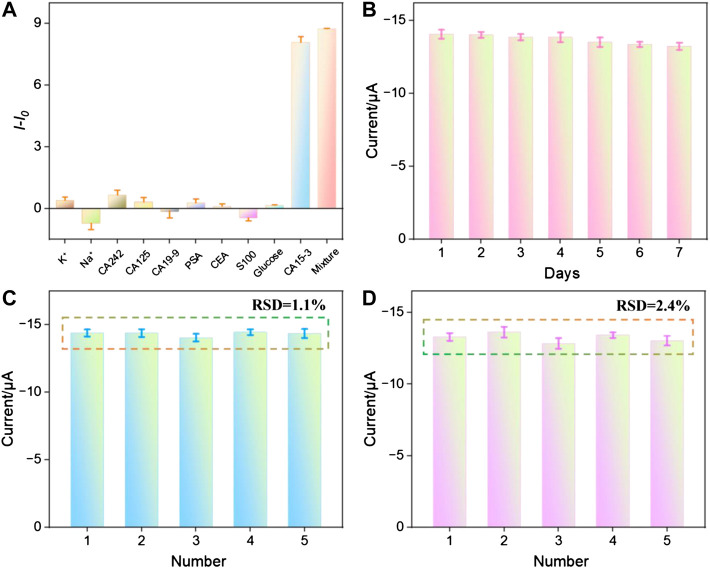
**(A)** The change of peak current when the aptasensor was incubated with different substance. **(B)** The peak current measured when the aptasensor was stored for different time after incubation with CA15-3 (1 U/mL). **(C)** The peak current obtained in successive measurements after the aptasensor was incubated with CA15-3 (1 U/mL). **(D)** The peak current obtained on different aptasensors fabricated parallelly after they were incubated with CA15-3 (1 U/mL).

To assess the selectivity of the fabricated sensor, the reduction peak signal of the aptasensor before (*I*_0_) and after (*I*) incubation with possible interfering agents, including potassium ion (K^+^), sodion (Na^+^), carbohydrate antigen 242 (CA242), cancer antigen 125 (CA125), carbohydrate antigen 19–9 (CA19-9), prostate-specific antigen (PSA), carcinoembryonic antigen (CEA), S100, glucose, CA15-3, and their mixtures were measured. As shown in Figure 6A, the changes in the reduction peak signal (*I* − *I*_0_) only significantly increased when incubated with CA15-3 or the mixture containing CA15-3. This demonstrated that the sensor exhibited excellent selectivity. As shown in Figure 6B, when the sensor was stored at 4°C for 1 week, the electrochemical signal measured after incubation with 1 U/mL of CA15-3 still remained 94.7% of the initial measurement, indicating good storage stability. The peak current obtained in successive measurements was investigated. After the aptasensor was incubated with CA15-3 (1 U/mL), the reduction peak signal of five successive measurements displayed a relative standard deviation (RSD) of 1.1%, indicating high signal stability (Figure 6C). When five aptasensors were fabricated parallelly and applied for detection of CA15-3 (1 U/mL), the reduction peak current exhibited a RSD of 2.4%, indicating high reproducibility (Figure 6D).

### 3.8 Real sample analysis

The capability of the constructed aptasensor in real sample analysis was assessed using the standard addition method. The detection of CA15-3 in fetal bovine serum was presented in Table 2. It can be observed that the sensor exhibits good spike recovery (95.2%–107%) and low relative standard deviation (RSD, 1.1%–1.9%) for CA15-3 detection, indicating excellent accuracy in the analysis.

**TABLE 2:** Determination of CA15-3 in fetal bovine serum using the developed aptasensor.

Sample	Added (U/mL)	Found (U/mL)	Recovery (%)	RSD (%, n = 3)
serum^a^	0.0100	0.00952	95.2	1.9
	1.00	1.07	107	1.9
	10.0	9.56	95.6	1.8
	100	97.4	97.4	1.1

^a^ The fetal bovine serum was diluted by a factor of 50 using PBS (0.01 M, pH 7.4).

## 4 Conclusion

In summary, a bipolar silica nanochannel array film (bp-SNA) with two independent functional domains including nanochannels and an outer surface was employed for the immobilization of recognition ligands and electrochemical redox probes, leading to a probe-integrated aptasensor for reagentless electrochemical detection of CA15-3. The bp-SNA comprised a negatively charged inner layer (n-SNA) and a positively charged outer layer (p-SNA), that were sequentially grown on a supporting electrode using convenient process. The amino groups on the outer surface of bp-SNA were functionalized with aldehyde groups for covalent immobilization of recognition ligands. Electrochemical redox probes were anchored within the nanochannels of bp-SNA, enabling reagentless electrochemical detection. The immobilized electrochemical probes experienced electrostatic attraction from n-SNA and electrostatic repulsion from p-SNA, resulting in high probe stability due to these dual forces. The constructed aptasensor demonstrated electrochemical detection of CA15-3 with low detection limit and selectivity. Given the simplicity of the substrate electrode, ease of bp-SNA preparation, and the stability of the probes, the developed sensor holds potential applications in tumor biomarker detection.

## Data Availability

The original contributions presented in the study are included in the article/Supplementary material, further inquiries can be directed to the corresponding authors.
